# Exposures to Air Pollution and Noise from Multi-Modal Commuting in a Chinese City

**DOI:** 10.3390/ijerph16142539

**Published:** 2019-07-16

**Authors:** Yisi Liu, Bowen Lan, Jeff Shirai, Elena Austin, Changhong Yang, Edmund Seto

**Affiliations:** 1 Department of Environmental and Occupational Health Sciences, School of Public Health, University of Washington, 1959 NE Pacific Street, Seattle, WA 98195, USA; 2 Department of Epidemiology, Biostatistics and Occupational Health, McGill University, 1020 Pine Avenue West, Montreal, QC H3A 1A2, Canada; 3 Institute for Public Health and Information, Sichuan Center for Diseases Control and prevention, #6 Zhongxue Road, Chengdu 610041, China

**Keywords:** multi-modal commuting, traffic related air pollution, noise, personal monitoring

## Abstract

Background: Modern urban travel includes mixtures of transit options, which potentially impact individual pollution exposures and health. This study aims to investigate variations in traffic-related air pollution and noise levels experienced in traffic in Chengdu, China. Methods: Real-time PM_2.5_, black carbon (BC), and noise levels were measured for four transportation modes (car, bus, subway, and shared bike) on scripted routes in three types of neighborhoods (urban core, developing neighborhood, and suburb). Each mode of transportation in each neighborhood was sampled five times in summer and winter, respectively. After quality control, mixed effect models were built for the three pollutants separately. Results: Air pollutants had much higher concentrations in winter. Urban Core had the highest PM_2.5_ and BC concentrations across seasons compared to the other neighborhoods. The mixed effect model indicated that car commutes were associated with lower PM_2.5_ (−34.4 μg/m^3^; 95% CI: −47.5, −21.3), BC (−2016.4 ng/m^3^; 95% CI: −3383.8, −648.6), and noise (−9.3 dBA; 95% CI: −10.5, −8.0) levels compared with other modes; subway commutes had lower PM_2.5_ (−11.9 μg/m^3^; 95% CI: 47.5, −21.3), but higher BC (2349.6 ng/m^3^; 95% CI: 978.1, 3722.1) and noise (3.0 dBA; 95% CI: 1.7, 4.3) levels than the other three modes of transportation. Conclusion: Personal exposure to air pollution and noise vary by season, neighborhood, and transportation modes. Exposure models accounting for environmental, meteorological, and behavioral factors, and duration of mixed mode commuting may be useful for health studies of urban traffic microenvironments.

## 1. Introduction

Urban mobility is changing. With economic growth and urbanization, individuals are no longer constrained to a single transportation mode. Commuting can include private, public, and shared options, physically active and non-active options, as well as combinations of mode types, such as walking, bicycling, and trips by car, bus, light rail, and train. Changes in urban mobility can potentially alter numerous population health determinants through physical activity levels, stress, access to resources, transportation-related costs, and time, as well as exposures to air pollution, noise, and other environmental stressors [1,2,3,4]. Moreover, the impacts associated with mobility changes may differentially affect certain populations based on income and residential location [5,6,7,8]. Thus, access to commute options is becoming an important environmental and social justice issue [9].

Air pollution concentrations have been found to be disproportionately high in high-traffic cities around the world. In cities such as Hong Kong [10], Montreal, Toronto, and Vancouver [11], and Los Angeles [12], significantly higher pollutant exposures have been found in transport microenvironments. The Multi-Ethnic Study of Atherosclerosis study reported that in-vehicle exposure contributed 24% of participants’ ambient-source NO_2_ exposure on average in Winston-Salem and Los Angeles [13]. In a study from Europe, similar results have been found, where despite a small proportion of daily time being routinely spent on intra-city transit (usually no more than 1.5–2 hours per day), commuters can receive up to 30% of their inhaled daily dose of black carbon (BC), and approximately 12% of their daily PM_2.5_ personal exposure during their regular journeys [14]. In light of these findings and the absence of similar studies done in China, it is of great importance to estimate pollution exposures during transportation for Chinese cities. Transportation is also the major source of noise in cities [15]. For example, the measured time-weighted noise levels for the subway in New York city reached 80 decibels (dBA) to 90 dBA, with peaks of 106 dBA [16]. Both air pollution and noise has been related to various adverse health effects in humans. Previous studies document various adverse health effects of air pollution, including respiratory disease, cardiovascular systems, and birth outcomes. Also, excessive noise exposures has been related to annoyance, sleep disturbance, cardiovascular diseases, mental disorder, and children’s cognition. Thus, transportation has significant effects on the environment and health.

Personal pollution intake is related to commuting choices due to variations in exposure levels in microenvironment, minute ventilations, as well as commuting time. For example, biking is oftentimes recommended as an active commuting mode and non-emission choice. However, cyclists may experience increased air pollution exposure because of their higher minute ventilation. To date, available evidence linking pollution exposures and health effects for different commuting choices is limited. Understanding the relationship between the choices commuters make and their exposures may be key to reducing exposures by informing behavior and adapting urban infrastructure.

Chengdu is a megacity in the Sichuan Basin, southwest China, with a 2016 population of around 14 million. As the capital of Sichuan province, Chengdu is the only megacity in the western part of China, with 72% of it being urbanized [17]. Located in the bottom of the Sichuan Basin, air pollution is a major concern because the province suffers from acidic rain and frequent haze events due to low wind speed and relatively high humidity [18]. The topography surrounding Chengdu, with Longquan Mountain to the east and Qionglai Mountain to the west, hinders the dispersion of locally produced pollutants and causes high levels of pollution, especially under certain weather conditions [19].

To date, a few studies have measured traffic-related air pollution in Beijing and Shanghai. However, none of these have characterized the potential for differences in noise exposures by transit mode and exposures between different commuting neighborhoods. With rapid development in recent decades, Chengdu has been experiencing changes in urban mobility options as populations confront traffic congestion. The city operates mature private and public transportation systems. Car ownership and private car use have grown considerably. As one indicator of the growth, according to the Traffic Management Bureau of the Public Security Ministry of China, the car ownership in Chengdu witnessed a ten-fold increase in the last decade, reaching 4.94 million at the end of 2017, and second only to Beijing in terms of mainland cities in China [20]. For public transportation, the Chengdu Public Transport Group Co., Ltd., reported that Chengdu had a total of 668 bus routes, with more than 1.5 billion passenger trips having been taken in 2017 [21]. The metro system, introduced in 2010, now includes 6 lines. The nearly 200-km length system served 2.14 million passenger trips on average per day in 2017 [22]. Shared bicycle use has also been booming in Chinese cities since its introduction in 2014. Formerly called the “kingdom of bicycles,” Chengdu is a bike-friendly city because of its flat terrain. With more than 1.3 million shared bikes in the city, Chengdu ranks first according to reports from both Mobike and Ofo—two of the biggest companies in the Chinese shared bicycle market—with the longest average distance of 2.3 km per ride and the highest riding index (a combined indicator of riding distance and time per user) compared to other Chinese cities. Although the city has a large population and an extensive and complex traffic system, transit-related noise and air pollution exposures have not been well characterized in Chengdu.

The current study aimed to characterize variations in personal traffic-related pollution exposures (PM_2.5_, black carbon (BC), and noise) for different transportation modes and different neighborhoods using scripted trips in summer and winter in Chengdu.

## 2. Methods

### 2.1. Neighborhood, Routes and Modes

**Figure 1:**
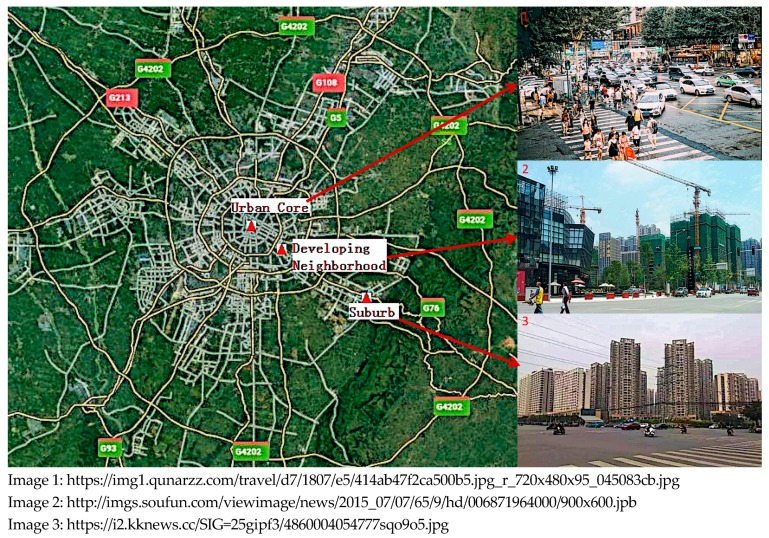
Map of the three sampling neighborhoods in Chengdu City.

Chengdu is the capital city of Sichuan province in Southwest China. The weather in the city is humid (with annual average of relative humidity around 80%). The dominant wind direction in Chengdu is Northeast, with daily average wind speed of 1.1 m/s to 1.6 m/s. Similar to other major cities in China, Chengdu consists of five ring roads (from the inner city to the outside are named as the first ring road, second ring road, and up to the fifth ring road) that divide the city in terms of land use and population density. In Chinese cities, the oldest and densest areas of the city typically lie within the first few ring roads, and the addition of ring roads over time reflect the growth of the city, with suburban areas developed principally for residences or new businesses. From Chengdu’s city center to the suburbs, three representative neighborhoods were chosen for comparison: a neighborhood in the central city (Urban Core), one between the second and third ring road (Developing Neighborhood), and another outside the fourth ring road (Suburb) (Figure 1). The Urban Core is the economic center and traffic hub of the city, where shopping malls and office blocks are clustered. The Developing Neighborhood is an example of a historically industrial area that was outside of the city, but is now in transition from an area that previously supported a dismantled steel plant to a new residential neighborhood. With high traffic flow on the second ring road, which is a major urban thoroughfare, there are also several construction sites along the road, which reflects the development that is occurring in this neighborhood. The selected Suburb is located in the center of Longquan district. It covers the southeast suburban area of Chengdu, and is the east entrance to the city, with a population of 0.66 million in 2016. Although it is principally a residential area, truck traffic is not unusual during the day, although trucks are not allowed to enter the third ring road between 7 am and 10 pm.

Four modes—car, bus, subway, and shared bike—were sampled within each neighborhood as they represent the four main transportation options in Chengdu. One main travel route was chosen for each of the three neighborhoods (three routes in total). The selection criteria for the routes considered:

(a) Identifying a route that was approximately 2–3 km in distance. To make each mode of transportation feasible and based on the reported average distance per ride of the shared bike of 2.3 km [23], the three routes were designed to be no more than 3 km in distance.

(b) The four trip modes were possible for each route. To make the exposure measures comparable between modes, and not entirely due to routing differences, all modes were ensured to be possible for each route.

The final selected scripted routes were: in the Urban Core, a route from the People’s Park subway station to the Chunxi Road subway station (2.4 km); in the Developing Neighborhood, a route from the Tazishan Park subway station to the Dongda Road subway station (2.8 km); and in the chosen Suburb, a route from the Longping Road subway station to the Longquan subway station (2.1 km).

### 2.2. Exposure Measurements

For each of the three neighborhood routes, trips were repeated by research staff in both the summer (August 2017) and winter (December 2017) seasons. For each season, five trips were conducted for each of the four modes (i.e., 20 trips in each season for each neighborhood route; 5 trips × 4 modes × 2 seasons × 3 neighborhood routes = 120 trips in total). The trips in each neighborhood included both weekdays and weekends, morning and afternoon, and rush hour as well as off-peak hours. All the routes were travelled in both directions between 9 am and 4 pm. In the summer and winter sampling campaigns, noise levels and air pollution concentrations were simultaneously measured by portable monitors. For safety, and to make it easier to manage multiple instruments, each trip was monitored by two to four researchers traveling together, carrying different instruments. PM_2.5_ was measured by an optical particle counter, the Portable University of Washington Particle (PUWP) monitor (University of Washington, Seattle, WA, USA), which collects 12 readings per minute (an estimate of PM_2.5_ mass concentration and particle count concentrations for 6 size bins from 0.3 to 10 um based on the volume-fixed chamber); BC was monitored by the microAeth AE51 (AethLabs, San Francisco, CA, USA), set at a flow rate of 100 ml/min and time base of 30 seconds (30 seconds per reading); and noise level was recorded by the NoisePro DLX Dosimeter (3M, St. Paul, MN, USA), with 1-second measurements. Research staff also carried a Bluetooth GPS data logger BT 335 (GlobalSat, Chino, CA, USA) during each trip in order to record the start and end times and to confirm the actual travel route for each trip. Before the monitoring campaign, the flow rate of AE 51 was checked, which was off by less than 1%. Thus, no additional flow calibration was performed for the AE51. Additionally, before and after each trip, the NoisePro DLX dosimeter was calibrated against the Quest QC-20 calibrator (3M, St. Paul, MN, USA). Clocks in all of the instruments were synchronized before sampling campaigns, and inlets for the instruments were fixed on the shoulder or the backpack strap of research staff to measure breathing zone concentrations. For subway trips, exposures were delineated as the period from entering a subway station entrance to leaving the exit at a subsequent station; and similarly, for each bus trip, from the arrival at the start bus station to the exit at the subsequent end station. After each trip, measurements were immediately downloaded from all instruments and safely archived as separate files for later data analyses. After the data extraction, the BC data was checked for the flow, and both the PM_2.5_ and BC data was checked for zero readings to detect any instrumental failure during the monitoring.

### 2.3. Statistical Analysis

Recorded data for PM_2.5_, BC, and noise levels for each trip were extracted from the raw data files in each instrument. Multiple quality control measures were utilized to evaluate the raw data files to ensure an acceptable level of data quality. First, an optimized noise-reduction algorithm (ONA) was used to reduce noise in real-time BC data obtained, which accounts for changes in sensitivity of the measurement related to changes in filter attenuation [24]. The ONA BC data were used in subsequent analyses. Observations with missing values for any pollutant were also removed (25 missing data points for PM_2.5_, 208 missing data for BC, and no missing data for noise). PM_2.5_ measurements with corresponding PM_0.3_ counts equal to 0 were considered unreliable and were removed (353 data points were removed out of 16,543). Finally, PM_2.5_ measurements larger than 1,000 μg/m^3^ and BC measurements larger than 100,000 ng/m^3^ were considered outliers and were removed as well (18 outliers for PM_2.5_, and 6 outliers for BC). After the quality control processes, 1-minute averages of PM_2.5_ were computed from the PUWP monitor data. All of the exposure data were then stratified by season, neighborhood, mode of transportation, days of the week (workdays/weekends), and hours of the day (data was grouped into 2-hour chunks as 9:00–10:59, 11:00–12:59, 13:00–14:59, 15:00–16:59, and 17:00–18:59) for descriptive analysis. One-minute average data were collected for all three pollutants and merged for each minute of sampling to calculate pairwise Spearman’s correlation coefficients.

With repeated measurements and different background pollutants levels in each trip, linear mixed models were applied to explain variations in each traffic-related pollution by season, neighborhood, and travel modes. Separate multivariable mixed effect regression models were estimated with each single pollutant as the dependent variable (1-minute average PM_2.5_, 30-second BC, and 1-second noise), with season, neighborhood, and mode as fixed effects independent variables, and with trip as a random effect (random intercept). Days of the week (workdays and weekend) and hours of the day (grouped into 2-hour chunks) were also added into models as fixed effects to adjust for various pollutant distributions in different days and hours. Additional single-pollutant multivariable linear mixed models were established to account for potential interactions between transportation modes and different types of neighborhoods. All data processing and statistical analyses were performed in R 3.5.2 (http://www.R-project.org/) (R Foundation for Statistical Computing, Vienna, Austria).

## 3. Results

**Figure 2:**
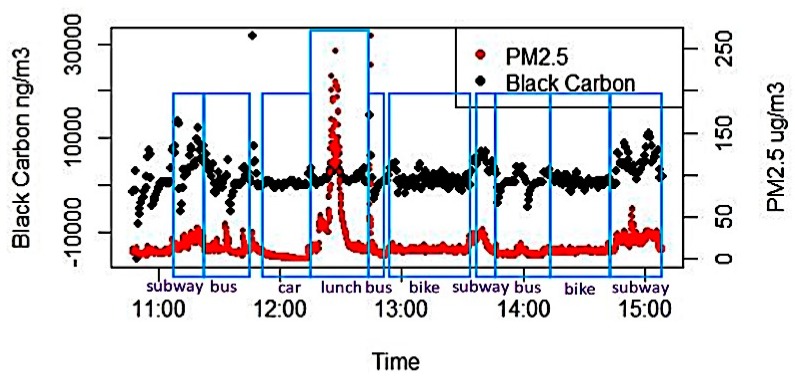
Time-series plot for PM_2.5_ and BC raw data (before ONA smoothing) in one day.

A time-series plot for PM_2.5_ and BC raw data (raw measurement before ONA smoothing) for a single day is shown in Figure 2 and shows the variations in different exposures that commuters experience as they transition between transportation modes within a multi-modal trip. Looking at some of the demarcated modes (e.g., bus and subway), the exposures are not constant, but can vary during an entire trip, and within modes of the trip. Also, the high levels of PM_2.5_ observed during lunch (not during a trip) illustrates the potential importance of exposures that occur in other microenvironments, and suggests that certain indoor microenvironments can contribute substantially to cumulative PM exposure. Furthermore, the figure shows that the correlations that may exist between PM_2.5_ and BC are not immediately obvious from a single day of monitoring, even with repeated measures. Furthermore, it is noteworthy that the BC raw data measured by the aethalometer was noisy, with many negative values. Thus, all the statistical analyses were based on ONA smoothed BC data.

Descriptive statistics of the three pollutants based on all 120 trips across the summer and winter seasons are summarized in Table 1. PM_2.5_ and BC had much higher concentrations (*p* < 0.05) in the winter than the levels in summer (PM_2.5_ median: 123 vs. 33.6 μg/m^3^, BC median: 8916.5 vs. 1896.3 ng/m^3^); however, noise levels were slightly higher in the summer time (within and across neighborhoods). In terms of the spatial distribution, the urban core neighborhood had the highest median PM_2.5_ and BC concentrations across seasons. In the summer time, the suburban area had the lowest PM_2.5_ and BC levels, while in the winter time, the lowest PM_2.5_ and BC levels were recorded in the developing neighborhood. Noise levels were roughly the same in the three neighborhoods (within and across seasons). Since days of the week and hours of the day have great impacts on the distribution of pollutants, the data was also summarized by workday/weekend and hour of the day as well (Table S1). All the pollutants (PM_2.5_, BC, and noise) had higher levels on weekends than on workdays.

Table 2 summarizes the correlations between PM_2.5_, BC, and noise in different neighborhoods and seasons. All of the pollutants were positively correlated with each other, except for PM_2.5_ and noise measured in Developing Neighborhood in the winter time (ρ = −0.11) and BC and noise measurements in the Urban Core across seasons (r = −0.05). PM_2.5_ and BC were strongly correlated with each other in the urban core (r = 0.84) and suburban (r = 0.86) neighborhoods across seasons. PM_2.5_ and BC had a slightly stronger correlation in the summer (ρ = 0.67) than in the winter (ρ = 0.64) across neighborhoods. In general, noise had weak correlations (ρ <0.4) between PM_2.5_ and BC.

The three pollutants were observed to vary by modes of transportation (Table S2, Figure S1 in the Supplementary Materials). In the summer, riding a car exposed people to the lowest median PM_2.5_ (8.4 μg/m^3^), median BC (211.5 ng/m^3^), and mean noise levels (62.3 dBA), while riding a subway was the most polluted mode of transportation for all three pollutants (median PM_2.5_: 39.4 μg/m^3^, median BC: 7,809.4 ng/m^3^, mean noise: 76.1 dBA). In the winter time, biking had the highest median PM_2.5_ (179.0 μg/m^3^) and median BC (10,979.5 ng/m^3^) concentrations compared with the other three modes of transportation, while subway exposed people to the lowest median PM_2.5_ level (92.3 μg/m^3^), and riding a bus was the least polluted modes in terms of BC concentration (median of 7575.0 ng/m^3^). Riding a car and a subway still had the lowest (64.3 dBA) and highest (75.5 dBA) mean noise levels, respectively, in the winter time.

Estimates from multivariable linear mixed models adjusted for weekends and workdays and hours of the day are summarized in Table 3. Trips in the winter exposed travelers to higher PM_2.5_ and BC concentrations and lower noise levels. Controlling for season and neighborhood, riding a car resulted in lower PM_2.5_ exposures (−34.4 μg/m^3^; 95% CI: −47.5, −21.3), lower BC exposures (−2016.4 ng/m^3^; 95% CI: −3383.8, −648.6), and also decreased noise exposures (–9.3 dBA; 95% CI: −10.5, −8.0) compared to biking. Riding a subway exposed commuters to increased BC (2349.6 ng/m^3^; 95% CI: 987.1, 3722.1) and noise levels (3.0 dBA; 95% CI: 1.7, 4.3) compared to cycling. Taking a bus also exposed travelers to higher noise levels (1.8 dBA; 95% CI: 0.5, 3.0) than biking. Compared to traveling in the suburban area, commuting in the Urban Core increased PM_2.5_ and BC levels (PM_2.5_: 18.8 μg/m^3^; 95% CI: 6,5, 31.1; BC: 2939.4 ng/m^3^; 95% CI: 650.8, 3219.3). Differences in noise levels were not statistically significant between neighborhoods.

**Figure 3:**
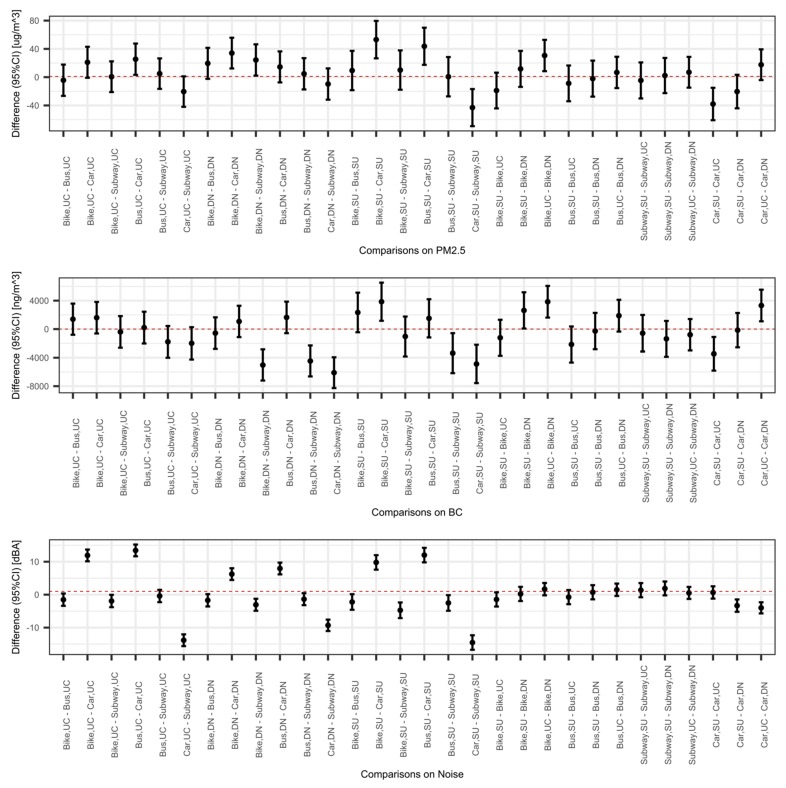
Pairwise comparisons for PM_2.5_, BC, and noise exposure during trips in mixed effect models with interaction terms between modes and neighborhoods. Abbreviations: UC represents Urban Core, DN represents Developing Neighborhood, SU represent suburb. Models were adjusted for season, days of the week (weekend/workdays). and hours of the day; The pairwise comparison is the contrast between groups, in which case, comparisons were computed between modes of transportation and neighborhoods. The x axis shows the specified comparison groups, the y axis is the difference between comparison groups. The point in the figure is the estimated difference in pollutant levels between groups, while the bar shows the 95% CI of the point estimate.

Estimates from multivariable linear mixed models with interactions between modes and neighborhoods are summarized in Figure 3 and Table S3 in the Supplementary Materials. The left half of Figure 3 shows the contrast of pollutant levels between modes in each neighborhood and the right half of the figure shows the contrast of pollutant levels between neighborhoods for each mode. The interaction term was only statistically significant for noise (*p* = 0.0007). After adding the interaction term into linear mixed models, the direction of main effects did not change compared to results from the mixed effect model without an interaction term between modes and neighborhoods. In the selected Suburb, riding a car had the lowest PM_2.5_ concentrations compared to biking, taking the subway, or a bus. In the Urban Core, taking a bus exposed individuals to 25.4 μg/m^3^ (95% CI: 3.3, 47.5) higher PM_2.5_ than riding a car. In the Developing Neighborhood, biking exposed travelers to higher PM_2.5_ concentrations than people using a car or the subway. Between neighborhoods, biking in the Urban Core had higher PM_2.5_ concentration than in the Developing Neighborhood, while riding a car had higher PM_2.5_ levels in the Suburb than in the Urban Core. For BC exposures, biking exposed commuters to higher BC levels in the Suburb and Developing Neighborhood. Traveling in the Urban core had similar BC levels for the four modes of transportation; also, taking a bus or the subway expose travelers to similar BC concentrations across different areas of the city. Noise levels of different modes of transportation have similar patterns in the three measured areas, riding a bus or a bike had the highest noise levels, while riding a car exposed commuters to the lowest noise levels. Biking, riding the subway, and riding a bus did not vary significantly between areas in the city; however, riding a car in the Developing Neighborhood was noisier than in the Urban Core and the Suburb.

## 4. Discussion

The choice of daily commuting can have important consequences on an individual’s exposure to traffic-related pollution. Personal exposure to PM_2.5_, BC, and noise in four transportation modes (biking, car, subway, and bus) were examined in summer and winter 2017 in Chengdu, China. A total of 120 trips were assessed to characterize traffic-related pollution in the megacity. Linear mixed models showed PM_2.5_ and BC levels in transportation were much higher in the winter time than the summer months, while noise levels were lower in the winter months. PM_2.5_ and BC concentrations varied spatially, and the Urban Core had the higher air pollution levels compared to other areas. All the pollutants varied between transportation modes. Commuters using a car exposed to lower PM_2.5_ and BC levels, while riding a subway had higher BC concentrations than other modes. Riding a car also exposed commuters to lower noise levels. However, PM_2.5_, BC, and noise exposures were not affected by neighborhood during bus and subway trips in Chengdu city.

PM_2.5_ and BC concentrations in different microenvironments showed large seasonal variation. Both of the two air pollutants levels were more than three times higher in the winter time than in the summer. Previous ambient air studies reported large seasonal variation of ambient air pollution levels in Chengdu. Wang et al. used 47-mm Teflon filters to collect PM_2.5_ samples, and observed the highest monthly mean PM_2.5_ concentrations in winter (113.5 ± 47.8 μg/m^3^) and the lowest in summer (45.1 ± 15.2 μg/m^3^) [25]. Shi et al. used medium-volume air samplers to measure PM_2.5_ and their components from January 2009 to March 2013, and found the concentration peak of PM_2.5_ and elemental carbon (or BC) was in winter (especially in January and December) [26]. Unfavorable meteorological conditions, such as low wind speeds, low mixing heights, and relatively low precipitation amount in Sichuan Basin, were common in the winter time in Chengdu [25]. The lower atmospheric boundary layer and large number of days with stagnant atmospheric conditions impair the transport and dispersion capacity of ambient air pollutants [27]. Compared to other provincial capitals, Chengdu has a relatively high fractional contribution to PM_2.5_ concentration from transportation (7.4%); however, the maximum daily fractional contributions from transportation did not present significant seasonal variations [28]. Thus, peaks of air pollution levels in traffic found in the winter time may be due to the higher ambient air pollution concentrations during those months.

Measured PM_2.5_ and BC concentrations also presented spatial variations. The urban core neighborhood had the highest PM_2.5_ and BC concentrations. The Urban Core is not only the economic center, but also a transportation center of the megacity. Similar to the ring-like urban planning in Beijing, Chengdu consists of five ring roads. All of the arterial roads meet in the urban core. Also, as the economic center, the Urban Core is filled with office buildings and a large number of commercial complexes. Thus, this area suffers from heavy traffic, especially during rush hours. The current study also showed that PM_2.5_ and BC concentrations were highly correlated (ρ = 0.84) in the Urban Core, which suggests that the source of the air particles in that area is likely traffic. Therefore, the large amount of vehicle exhaust emission in the Urban Core due to the urban planning in Chengdu may explain the high air pollution levels in this area. 

Unlike what is seen in the Urban Core, the Suburb is where many factories are located. Because of this, and the regulation in Chengdu that prohibits trucks from entering the city during the day (except for a very limited number of specific vehicles), the Suburb tends to have much more truck traffic than other neighborhoods. In China, most passenger cars use gasoline or compressed natural gas as fuel, while trucks typically use diesel as fuel, which results in higher emissions of PM. The current study found a strong correlation between PM_2.5_ and BC in the Suburb (ρ = 0.86), which supports the idea that higher truck traffic in that area contributes to the higher air pollution levels measured there. The Developing Neighborhood is still transitioning from an industrial to a residential area and has a lot of construction taking place. Since a very limited number of trucks are allowed to enter the city in the day, there is comparatively less traffic in the daytime. In light of this, the weaker (ρ = 0.55) correlation observed between PM_2.5_ and BC concentrations in the Developing Neighborhood is not surprising.

The linear mixed model results indicate that car passengers were exposed to lower air pollution levels. The observed low exposures in car is supported by previous studies. Huang et al. sampled PM_2.5_ for three commuting modes (taxi, bus, and bicycle) for 18 weekdays between December 2010 and February 2011 in Beijing. Using a portable aerosol spectrometer to measure real-time PM_2.5_ concentration in both heavy and light traffic, Huang et al. suggested that riding a taxi had the lowest average PM_2.5_ concentrations (31.6 μg/m^3^) compared to cycling (49.1 μg/m^3^) or riding a bus (42.4 μg/m^3^) [29]. Similarly, a study in Santiago, Chile, where a handheld optical particle counter was used to measure PM_2.5_ concentrations during travel on buses, bicycles, cars, and subways in both summer and winter, found that being in a car had the least impact on personal PM_2.5_ exposure [30]. One likely reason for the lowest PM_2.5_ levels found in cars is that most cars in China (as well as the car used in the sampling campaign) are newer designs, and the ventilation system of the car may make the in-cabin microenvironment cleaner.

Published studies have shown inconsistent results for air pollution associated with traveling by subway. A panel study in Taipei sampled PM_2.5_ on 120 young adults during 1-hour morning commutes between January and March in 2012–2014 for same three modes of transportation as the current Chengdu study (electrically powered subway system, gas-powered buses, gasoline-powered cars) and walking. Results of the Taipei study indicated that subjects were exposed to the lowest PM_2.5_ concentrations when using a subway (22.3 ± 6.9 μg/m^3^) compared to riding a car (29.2 ± 11.3 μg/m^3^), taking a bus (32.2 ± 12.4 μg/m^3^), or walking (42.1 ± 18.2 μg/m^3^) [31]. However, other studies found riding a subway had higher air pollution concentrations than other modes of transportation in Shanghai and New York [32,33]. Although all of the subway trains are newly designed (implemented in 2010), electrically powered, and the ventilation system is kept running inside both the subway station and the coach of the train, our study suggest highest BC concentrations during riding a subway than other modes. The source of pollution in subways may come from rails, wheels, catenaries, brake pads, pantographs [34], and brakes [35]. Also, the depth of the subway station and number of trains passing through the station could also help explain the differences seen in air pollution concentration in subways [33]. Additionally, magnetite and hematite resulting from the friction of the metal-to-metal contact between the car wheels and the rail can interfere with the measurement by the Aethalometer in the metro system. This may help explain the higher level of air pollution measured in the Chengdu Metro system.

Based on the mixed effect model for PM_2.5_ exposure, cycling in the urban core in winter months exposed people to the highest air pollution levels. Previously, it has been suggested that modes that come into close proximity to traffic would lead those travelers to experience higher exposures [36]. Huang et al. found in Shanghai that cycling had the highest average PM_2.5_ concentrations (49.1 μg/m^3^) compared to riding a bus (42.4 μg/m^3^) or a taxi (31.6 μg/m^3^) [29]. A panel study in Taipei also showed subjects who walked were exposed to the highest air pollution concentration (PM_2.5_: 42.1 ± 18.2 μg/m^3^) in the winter sampling campaign [31]. Chaney et al. examined personal PM_2.5_ exposures in the summer on a single 2.7-km arterial urban route in Salt Lake City during rush hour. They found that higher PM_2.5_ exposures occurred while biking, walking, and taking a bus compared to riding a window-closed car or the light rail [37]. Biking in the winter exposes people directly to the high ambient air pollution levels and vehicle exhaust emissions, while the ventilation and filtration system of the vehicles reduce the penetration of air pollutants from the outside. Aside from the possibility of direct exposure to high air pollution levels during cycling, people tend to have higher inhalation rate when biking and would intake increased amount of air pollutants. Thus, biking during highly polluted days or in polluted areas could increase personal intake of air pollutants compared to vehicle transportation and may have the highest potential to affect health in the city. Therefore, more research is needed to determine the tradeoffs between cardiovascular health benefits from cycling versus the harm from pollution exposure in Chengdu.

Riding a car was found resulted in lower noise exposures than the other transportation modes. Car travel is an almost-closed microenvironment compared to the other three modes of transportation, and newly designed cars generally incorporate sound insulation. Thus, riding a car may be less noisy than the other commuting modes. The current study’s measurements showed subway and bus trips were noisier, which is inconsistent with some previous studies. Studies in Taipei and Europe indicated that walking and biking to be the noisiest mode of transportation. [31,38]. The inconsistency between our study and previous ones may be due to the differences in rail and wheel design, as well as sound insulation design of the bus and Metro system. Additionally, the subway and bus in Chengdu were usually at full passenger capacity when noise measurements were taken. Higher passenger exchange rate during commuting, as well as the larger number of passengers in the microenvironment observed in the Chengdu study, may explain the difference between our study and others. Our study also found slightly higher noise levels in the summer compared to the winter. Chengdu is humid in summer and many insects can be found in the city, such as cicadas. Cicadas are a major source of ambient noise aside from traffic during the summer time monitoring campaign by field staff. Both acute and chronic noise exposure have been associated with adverse health outcomes, including hearing loss, annoyance, sleep disturbance, cardiovascular diseases, and cognitive impairment (mainly in children) [39]. Other investigators have found increased noise exposure is associated with arousals of the autonomic nervous system and endocrine system, increased systolic and diastolic blood pressure, changes of heart rate, and causes the release of stress hormones [40,41]. Meta-analyses have determined associations between transportation noise and cardiovascular diseases, with observed thresholds for the exposure–response link of different diseases ranging from 40 to 60 dBA [42,43]. The average noise levels found in this current study for different modes of transportation all exceeded 60 dBA. As a known risk factor, noise levels should be monitored to evaluate the health effect in commuter studies, and also in health effect analysis of traffic-related air pollution to tease out potential confounding effects of noise.

It is noteworthy that published exposure studies on traffic-related air pollution have various inconsistencies. Aside from modes of transport, other factors potentially influencing the assessment of personal exposure in traffic include measurement factors (e.g., pollutants measured, position of the measurements in relation to the breathing zone), personal or individual factors (e.g., breathing rate, personal behavior or choices, and personal sources), traffic factors (e.g., traffic count and type, traffic flow, junction layout, link length, etc.), and meteorological factors (e.g., wind speed, wind direction, etc.) [36]. Additionally, personal exposure in different modes of transportation may also be related to the energy source of the vehicle, passenger population in the microenvironment, and ventilation and filtration systems that are in place in the vehicles. Differences in any of the above-mentioned factors may lead to inconsistent results between studies.

The world's population is estimated to reach 10 billion people by 2050, with 75% of this population living in cities [44]. At that time, 90% of the 2.5 billion more people expected to be in urban areas will be found in Asia and Africa [45]. Intra-city commuting potentially affects human health by choices in transportation modes, route, time through various air pollution, physical activity, climate factors, as well as interactions between these variables. The current study further analyzed the interaction between modes of transportation and commuting neighborhoods in mixed effect models. Statistical results showed different modes of transportation had similar BC concentrations in the urban core. Biking as an active mode of transportation has been recommended, but there is concern about the high air pollution concentrations present during biking. However, the current study’s findings suggest that in certain areas, different modes of transportation had similar pollution levels. This may potentially impact urban planning and policy decision-making to support active and public modes of travel in Chengdu. As mentioned previously, the developing neighborhoods are still under construction. Designing bike-friendly communities in this area would be suggested to promote physical activity and population health. The Urban Core has been confronted with traffic congestion for a long time. Promoting the use of public transportation in the urban core area would help reduce traffic emissions and solve traffic congestion issues being experienced there. Additionally, personal intake of air pollutants is also related to time of exposure and inhalation rate. This current study found lower air pollutant levels when riding a subway or a car. However, the time spent on the same route is generally shorter when traveling by subway than the other modes. This is especially true when traveling in urban areas during peak hours. In contrast, although, riding a car exposes a commuter to the lowest PM_2.5_, BC, and noise levels, it is not unusual to spend more time on the road because of traffic jam, particularly in the urban core. Thus, further studies are needed to fully consider pollutant exposure and transportation time to better recommend commuting choices to citizens.

Every study has limitations, and the current one is no exception. The main limitation of this study was the use of a limited number of scripted trips and neighborhoods. Thus, the generalizability of the study may be limited. Air concentrations were collected in the summer and winter of 2017 only, so no between-year variations could be examined. Aside from pollutants levels were only measured in the daytime, while no measurements were taken at night. Also, measurements were collected by research staff and we relied on scripted measurements for three specific routes, rather than taking samples from actual, unscripted routes used by real commuters. Nevertheless, the monitoring mimicked personal monitoring with repeated measurements, which captured several important characteristics of pollution during different modes of transportation. 

Although this study measured pollution levels under different situations, it did not address the cumulative exposure for each pollutant. The cumulative exposure, which accounts for total time of exposure, would be more relevant to understanding the health effects of exposures during intra-city commuting. Thus, further studies are needed to generalize personal air pollution exposure models in transportation by mode choice, commuting route and time, and meteorological factors, and to assess the potential health effects on urban populations.

## 5. Conclusions

The current study investigated personal exposures to PM_2.5_, BC, and noise experienced during trips in cars, buses, and subways, as well as during bicycling in Chengdu, China. All transportation modes utilized scripted and repeated routes in three different neighborhoods. The total of 120 trips was conducted across summer and winter seasons, and covered the mornings and afternoons of weekdays and weekends. The monitoring campaign showed personal PM_2.5_, BC, and noise exposures in traffic microenvironments varied by season, neighborhood, and modes of transportation. Air pollution levels in traffic were significantly higher in the winter than in the summer. Traveling in the urban core area of Chengdu resulted in higher air pollution levels. Riding a car resulted in lower PM_2.5_ concentrations. Taking a bus or the subway resulted in higher noise levels, while car trips had lower noise levels. However, in certain areas, PM_2.5_ and BC levels were not affected by trip mode, or had lower concentrations during active and public transportation. Riding a bike in the Urban Core during the winter months may have the highest potential to affect individual health in this city. In the future, exposure models that account for environmental, meteorological, and behavioral factors, as well as duration of commuting, are needed in health studies of urban traffic microenvironments.

## Figures and Tables

**Table 1:** Summary of exposures during trips by season and neighborhood.

Pollutants	Urban Core			Developing Neighborhood			Suburb			All Area		
	N ^1^	Median (Mean) ^2^	IQR (SD) ^3^	N ^1^	Median (Mean) ^2^	IQR (SD) ^3^	N ^1^	Median (Mean) ^2^	IQR (SD) ^3^	N ^1^	Median (Mean) ^2^	IQR (SD) ^3^
**Summer**												
PM_2.5_ (μg/m^3^)	246	38.4	11.8	337	38.7	15.1	243	10.9	15.3	826	33.6	26.3
BC (ng/m^3^)	581	2408.5	3616.9	471	1891.8	3486.8	477	895.1	3039.8	1529	1896.3	3570.2
Noise (dBA)	20171	72.8	7.7	14551	72.6	6.0	14489	72.8	6.7	49211	72.7	6.9
**Winter**												
PM_2.5_ (μg/m^3^)	370	178.2	79.8	289	91.0	25.0	185	157.0	97.5	844	123.0	98.0
BC (ng/m^3^)	1014	11979.0	8384.5	782	5139.0	4076.2	398	10227.0	6616.5	2194	8916.5	8348.2
Noise (dBA)	30291	72.3	6.3	23487	72.4	6.4	11953	72.8	7.5	65731	72.4	6.6
**All Seasons**												
PM_2.5_ (μg/m^3^)	707	95.0	144.1	535	63.0	52.6	428	47.5	130.2	1670	57.0	90.9
BC (ng/m^3^)	1595	8585.5	10996.5	1253	4162.5	5380.5	875	5062.0	9243.7	3723	5917.0	8662.2
Noise (dBA)	50462	72.5	6.9	38038	72.5	6.2	26442	72.8	7.0	114942	72.6	6.7

Note: ^1^ N is the number of measurements for each pollutant. ^2^ For PM_2.5_ and BC, the median was recorded; for noise, the mean was recorded. ^3^ For PM_2.5_ and BC, the interquartile rang (IQR) was recorded; for noise, the mean was recorded.

**Table 2:** Correlations between PM_2.5_, BC, and noise during trips by season and neighborhood.

**Summer**												
**Pollutants**	**Urban Core**			**Developing Neighborhood**			**Suburb**			**All Areas**		
	PM_2.5_	BC	Noise	PM_2.5_	BC	Noise	PM_2.5_	BC	Noise	PM_2.5_	BC	Noise
PM_2.5_	1	0.57	0.32	1	0.80	0.18	1	0.63	0.29	1	0.67	0.28
BC	-	1	0.25	-	1	0.34	-	1	0.35	-	1	0.39
Noise	-	-	1	-	-	1	-	-	1	-	-	1
**Winter**												
	**Urban Core**			**Developing Neighborhood**			**Suburb**			**All Areas**		
	PM_2.5_	BC	Noise	PM_2.5_	BC	Noise	PM_2.5_	BC	Noise	PM_2.5_	BC	Noise
PM_2.5_	1	0.51	0.03	1	0.12	−0.11	1	0.53	0.38	1	0.64	0.02
BC	-	1	0.16	-	1	0.33	-	1	0.18	-	1	0.16
Noise	-	-	1	-	-	1	-	-	1	-	-	1
**All seasons**												
	**Urban Core**			**Developing Neighborhood**			**Suburb**			**All Areas**		
	PM_2.5_	BC	Noise	PM_2.5_	BC	Noise	PM_2.5_	BC	Noise	PM_2.5_	BC	Noise
PM_2.5_	1	0.84	0.01	1	0.55	0.06	1	0.86	0.17	1	0.81	0.06
BC	-	1	−0.05	-	1	0.29	-	1	0.20	-	1	0.12
Noise	-	-	1	-	-	1	-	-	1	-	-	1

**Table 3:** Summary of multivariable mixed effects model results.

Variables	PM_2.5_ (μg/m^3^)			BC (ng/m^3^)			Noise (dBA)		
	Estimate	95% CI	SE	Estimate	95% CI	SE	Estimate	95% CI	SE
Intercept	51.7	37.8, 65.5	7.3	3834.5	2360.8, 5313.2	774.7	74.0	72.7, 75.2	0.6
Bike	reference			reference			reference		
Bus	−8.1	−21.4, 5.2	7.0	−895.9	−2268.9, 476.5	723.8	1.8 *	0.5, 3.0	0.7
Car	−34.4 *	−47.5, −21.3	6.9	−2016.4 *	−3383.8, −648.6	719.9	−9.3 *	−10.5, −8.0	0.6
Subway	−11.9	−25.1, 1.3	7.0	2349.6 *	978.1, 3722.1	723.2	3.0 *	1.7, 4.3	0.7
Suburb	reference			reference			reference		
Urban core	18.8 *	6.5, 31.1	6.5	1939.4 *	650.8, 3219.3	676.5	0.0	−1.1, 1.2	0.6
Developing Neighborhood	3.3	−9.1, 15.6	6.5	−114.1	−1407.3, 1177.6	680.5	0.3	−0.9, 1.5	0.6
Summer	reference			reference			reference		
Winter	110.9 *	108.3, 113.5	1.3	6251.6 *	5913.6, 6593.5	174.0	−1.3 *	−1.3, −1.2	0.0

Note: * Statistically significant based on the 95% CI; all the multivariable models were adjusted for neighborhood, season, days of the week (weekends/workdays), and hours of the day.

## Supplementary Materials

The following are available online at https://www.mdpi.com/1660-4601/16/14/2539/s1.
