# Supplementary material for: Exposures to Air Pollution and Noise from Multi-Modal Commuting in a Chinese City
Source: Int J Environ Res Public Health. 2019 Jul 16;16(14):2539. doi: 10.3390/ijerph16142539 (PMC6679126; doi:10.3390/ijerph16142539)
Supplement: Supplementary file 1 [file ijerph-16-02539-s001.pdf]

**Table S1** Summary of exposures during trips by workday/weekend hour of the day.

| Hour of the day | PM2.5 (ug/m³) | | | BC (ng/m³) | | | Noise (dBA) | | |
|---|---|---|---|---|---|---|---|---|---|
| | N[1] | median (mean)[2] | IQR (SD)[3] | N[1] | median (mean)[2] | IQR (SD)[3] | N[1] | median (mean)[2] | IQR (SD)[3] |
| **Workdays** | | | | | | | | | |
| 9:00am-10:59am | 73 | 97.0 | 92.4 | 186 | 8283.0 | 3543.4 | 6538 | 67.3 | 7.7 |
| 11:00am-12:59pm | 520 | 43.7 | 124.6 | 1180 | 6139.0 | 8755.2 | 36532 | 72.6 | 6.8 |
| 13:00pm-14:59pm | 389 | 33.0 | 34.9 | 812 | 2454.2 | 5473.2 | 24634 | 73.5 | 5.8 |
| 15:00pm-16:59pm | 84 | 68.8 | 22.9 | 321 | 3722.5 | 3160.0 | 9596 | 71.6 | 6.0 |
| 17:00pm-18:59pm | 11 | 147.0 | 7.5 | 62 | 10388.5 | 6258.8 | 1832 | 72.1 | 6.7 |
| **Weekends** | | | | | | | | | |
| 9:00am-10:59am | 58 | 169.0 | 98.2 | 116 | 12924.0 | 5873.5 | 3501 | 66.5 | 8.6 |
| 11:00am-12:59pm | 183 | 213.0 | 50.2 | 370 | 16710.5 | 6347.8 | 11251 | 74.4 | 5.0 |
| 13:00pm-14:59pm | 173 | 52.2 | 74.3 | 323 | 6435.0 | 7768.5 | 10347 | 73.9 | 6.3 |
| 15:00pm-16:59pm | 155 | 96.0 | 17.5 | 306 | 5271.0 | 4472.4 | 9269 | 72.9 | 6.9 |
| 17:00pm-18:59pm | 24 | 84.5 | 13.2 | 47 | 7685.0 | 9960.2 | 1442 | 77.9 | 5.0 |

1. N is the number of measurements for each pollutant
2. For $PM_{2.5}$ and BC, the median was recorded; for noise, the mean was recorded
3. For $PM_{2.5}$ and BC, the interquartile rang (IQR) was recorded; for noise, the mean was recorded

**Table S2.** Summary of pollutants levels in four modes of transportation.

| Modes | PM2.5 (ug/m³) | | | | BC (ng/m³) | | | | Noise (dBA) | | | |
|---|---|---|---|---|---|---|---|---|---|---|---|---|
| | mean | SD | median | IQR | mean | SD | median | IQR | mean | SD | median | IQR |
| **Summer** | | | | | | | | | | | | |
| Bike | 31.6 | 14.6 | 36.6 | 29.6 | 2637.7 | 4537.6 | 1674.0 | 2372.5 | 72.1 | 4.7 | 71.7 | 5.6 |
| Bus | 30.7 | 14.6 | 32.4 | 20.3 | 1844.2 | 2755.6 | 1667.0 | 2094.0 | 75.2 | 5.4 | 75.7 | 6.7 |
| Car | 13.8 | 13.2 | 8.4 | 22.4 | 435.6 | 1767.0 | 211.5 | 716.3 | 62.3 | 6.8 | 62.1 | 8.5 |
| Subway | 39.4 | 11.5 | 43.1 | 16.1 | 7809.4 | 4527.4 | 7616.0 | 5730.0 | 76.1 | 4.7 | 75.6 | 6.3 |
| **winter** | | | | | | | | | | | | |
| Bike | 171.9 | 54.8 | 179.0 | 103.0 | 11335.9 | 6435.4 | 10979.5 | 9355.0 | 72.8 | 5.3 | 72.5 | 6.8 |
| Bus | 141.9 | 54.4 | 117.5 | 108.0 | 8881.6 | 6005.9 | 7575.0 | 6188.0 | 73.3 | 6.0 | 73.0 | 7.7 |
| Car | 139.1 | 41.5 | 130.0 | 58.0 | 10167.3 | 3823.2 | 9440.0 | 6126.0 | 64.3 | 5.9 | 64.2 | 7.2 |
| Subway | 105.5 | 39.5 | 92.3 | 53.3 | 9574.6 | 5725.7 | 9604.5 | 8260.3 | 75.5 | 5.4 | 75.0 | 7.6 |

**Table S3.** Pairwise comparison for three pollutants in mixed effect models with interaction between modes and neighborhoods

| | Comparison | PM2.5 ($p_{interaction}$ = 0.908) | | | BC ($p_{interaction}$ = 0.408) | | | Noise ($p_{interaction}$ = 0.0007) | | |
|---|---|---|---|---|---|---|---|---|---|---|
| | | Estimate | SE | p.value | Estimate | SE | p.value | Estimate | SE | p.value |
| Urban Core | Bike - Bus | -4.4 | 11.3 | 0.7003 | 1392 | 1117 | 0.2126 | -1.52 | 0.96 | 0.1123 |
| | Bike - Car | 21 | 11.2 | 0.0645 | 1600 | 1132 | 0.1578 | 11.91 | 0.91 | <.0001* |
| | Bike - Subway | 0.6 | 11.1 | 0.9545 | -395 | 1134 | 0.7276 | -1.92 | 0.96 | 0.0444* |
| | Bus - Car | 25.4 | 11.3 | 0.0267* | 208 | 1139 | 0.8553 | 13.43 | 0.91 | <.0001* |
| | Bus - Subway | 5 | 11 | 0.6503 | -1787 | 1134 | 0.1151 | -0.41 | 0.96 | 0.6713 |
| | Car - Subway | -20.4 | 11 | 0.0663 | -1995 | 1155 | 0.0842 | -13.83 | 0.91 | <.0001* |
| Developing Neighborhood | Bike - Bus | 19.5 | 11.2 | 0.0858 | -565 | 1131 | 0.6174 | -1.71 | 0.96 | 0.0745 |
| | Bike - Car | 34 | 11.1 | 0.0031* | 1075 | 1124 | 0.3391 | 6.24 | 0.91 | <.0001* |
| | Bike - Subway | 24.3 | 11.3 | 0.0347* | -5028 | 1114 | <.0001* | -3.07 | 0.93 | 0.001* |
| | Bus - Car | 14.5 | 11.2 | 0.1975 | 1640 | 1131 | 0.147 | 7.94 | 0.91 | <.0001* |
| | Bus - Subway | 4.8 | 11.3 | 0.6737 | -4463 | 1106 | 0.0001* | -1.36 | 0.93 | 0.1432 |
| | Car - Subway | -9.7 | 11.3 | 0.3902 | -6103 | 1104 | <.0001* | -9.31 | 0.89 | <.0001* |
| Suburb | Bike - Bus | 9.4 | 14.2 | 0.5099 | 2336 | 1420 | 0.0999 | -2.22 | 1.21 | 0.0663 |
| | Bike - Car | 53.1 | 13.5 | 0.0002* | 3848 | 1368 | 0.0049* | 9.79 | 1.13 | <.0001* |
| | Bike - Subway | 10 | 14.2 | 0.4832 | -1036 | 1427 | 0.4681 | -4.74 | 1.21 | 0.0001* |
| | Bus - Car | 43.7 | 13.4 | 0.0017* | 1512 | 1370 | 0.2697 | 12.01 | 1.13 | <.0001* |
| | Bus - Subway | 0.6 | 14.2 | 0.9645 | -3371 | 1429 | 0.0183* | -2.52 | 1.21 | 0.0375* |
| | Car - Subway | -43.1 | 13.4 | 0.002* | -4883 | 1371 | 0.0004* | -14.53 | 1.13 | <.0001* |
| Bike | SU - UC | -18.9 | 12.9 | 0.1468 | -1215 | 1287 | 0.3449 | -1.45 | 1.1 | 0.1861 |
| | SU - DN | 11.7 | 13 | 0.3695 | 2625 | 1300 | 0.0435* | 0.22 | 1.1 | 0.842 |
| | UC - DN | 30.6 | 11.3 | 0.0084* | 3841 | 1136 | 0.0007* | 1.67 | 0.96 | 0.081 |
| Bus | SU - UC | -8.8 | 12.9 | 0.4974 | -2159 | 1288 | 0.0936 | -0.75 | 1.1 | 0.4954 |
| | SU - DN | -2.1 | 13 | 0.8734 | -276 | 1303 | 0.8323 | 0.73 | 1.1 | 0.5043 |
| | UC - DN | 6.7 | 11.3 | 0.5549 | 1883 | 1139 | 0.0983 | 1.48 | 0.96 | 0.1217 |
| Subway | SU - UC | -4.6 | 13 | 0.722 | -575 | 1308 | 0.6605 | 1.36 | 1.1 | 0.215 |
| | SU - DN | 2.3 | 12.7 | 0.857 | -1367 | 1282 | 0.2862 | 1.89 | 1.07 | 0.0786 |
| | UC -DN | 6.9 | 11.1 | 0.5339 | -793 | 1125 | 0.4813 | 0.53 | 0.93 | 0.573 |
| Car | SU - UC | -38 | 11.7 | 0.0017* | -3463 | 1202 | 0.004* | 0.67 | 0.95 | 0.4827 |
| | SU - DN | -20.4 | 12.1 | 0.0946 | -148 | 1232 | 0.9046 | -3.33 | 0.96 | 0.0005* |
| | UC - DN | 17.6 | 11.1 | 0.1176 | 3316 | 1134 | 0.0035* | -4 | 0.86 | <.0001* |

Abbreviations: UC represents Urban Core, DN represents Developing Neighborhood, SU represent suburb; Models were adjusted for days of the week (weekend/workdays) and hours of the day;
$p_{interaction}$ represents the p value for the interaction term in mixed effect models.
* represents $p < 0.05$

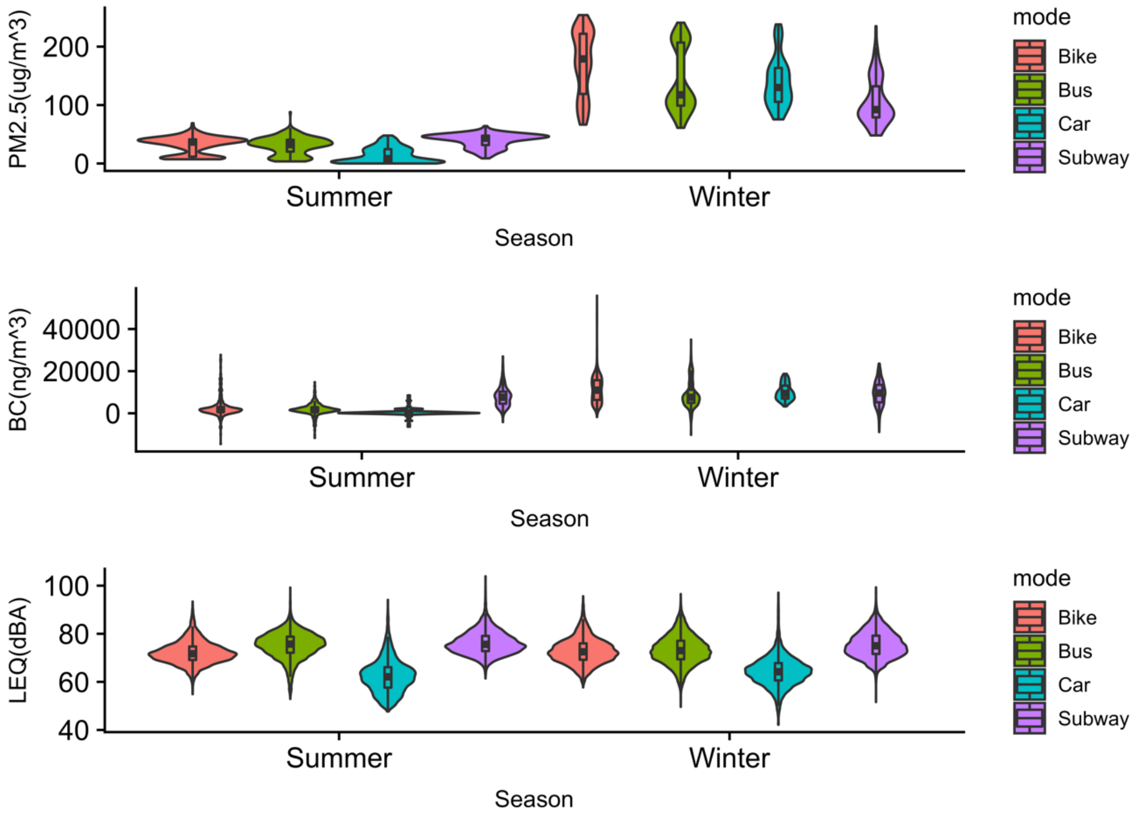


The violin plot includes all the sample points, with the width proportionate to the density of the data at different values. The box plot is also plotted upon the violin plot showing the median and interquartile range.

**Figure S1.** Violin plot for three-pollutant levels in the four modes of transportation
